# The prevalence of denture stomatitis in cigarette and hookah smokers and opium addicts: findings from Rafsanjan Cohort Study

**DOI:** 10.1186/s12903-021-01807-6

**Published:** 2021-09-17

**Authors:** Farimah Sardari, Parvin Khalili, Hamid Hakimi, Saadat Mahmoudaghaei, Pouya Abedi

**Affiliations:** 1 Department of Oral Medicine, School of Dentistry, Rafsanjan University of Medical Sciences, Rafsanjan, Iran; 2 Social Determinants of Health Research Centre, Rafsanjan University of Medical Sciences, Rafsanjan, Iran; 3 Non-Communicable Diseases Research Center, Rafsanjan University of Medical Sciences, Rafsanjan, Iran; 4 Student Research Committee, Rafsanjan University of Medical Sciences, Rafsanjan, Iran; 5 Student Research Committee, Rafsanjan University of Medical Sciences, Rafsanjan, Iran

**Keywords:** Denture stomatitis, Cigarette smoking, Opium use, Hookah smoking, Rafsanjan Cohort Study (RCS)

## Abstract

**Background:**

The aim of this study was to investigate the association of denture stomatitis prevalence with cigarette, hookah and opium consumption and also dose–response relationship between the cigarette smoking duration and odds of denture stomatitis in population of Rafsanjan cohort center.

**Methods:**

This cross-sectional study based on data of Rafsanjan Cohort Study (RCS) with 10,000 participants. After applying inclusion and exclusion criteria, 1619 participants were included in the analysis. Data were collected by oral examination and completion of pre-designed questionnaires to assess denture hygiene, smoking behavior, opium use and some other exposure variables. Multivariable logistic regression models were fitted to investigate possible association of cigarette, hookah and opium consumption and denture stomatitis.

**Results:**

Prevalence of denture stomatitis among all 1619 denture wearers was 21.6%. Cigarette smoking was associated with a higher odds of denture stomatitis, with the adjusted odds ratio (95% CI) of 2.29 (1.53–3.41). Also, dose–response increases were observed with the highest odds ratio in the 4th quartile for denture stomatitis (*p*-value < 0.001). Opium consumption was associated with a decreased odds of denture stomatitis (adjusted OR; 0.81, 95% CI 0.58–1.13) which was not statistically significant. Also interaction between opium consumption and cigarette smoking was not associated with higher odds of denture stomatitis (adjusted OR: 1.65, 95% CI 0.85–3.22).

**Conclusions:**

Based on the findings of the present study, while cigarette smoking had a dose–response relationship with the increased odds of denture stomatitis; this association was not found with opium consumption.

## Background

Denture stomatitis is a common clinical condition occurring in removable denture wearers. It is characterized by inflammation, erythema and edema of denture bearing mucosa which may accompanied by pain or burning [1]. Several studies reported that more than two thirds of denture wearers can develop denture stomatitis [2, 3]. The prevalence of this lesion in Iranian population was estimated to be 28.9% [4]. Although, the main etiology of denture stomatitis is not fully understood, some related risk factors have been identified including *Candida* infection, defects in the immune system, mucosal trauma, poor denture hygiene, and denture wearing pattern [2, 5]. As some of the risk factors are interrelated, they should be studied with multivariable analysis [5]. Although, many studies have been published on prevalence and etiology of denture stomatitis, the conclusion is still controversial [2, 3, 5, 6].

It is believed that most cases of denture stomatitis are associated with *Candida* growth [7] while literature reveals that cigarette smoking, as one of the local factors, either alone or with other factors can lead to *candida* infection [8, 9]. Smoking tobacco in other forms, such as hookah (also known as: water pipe, nargileh, shisha, ghalyan) indicated to be associated with suppressing the immune system cells, change the composition of the oral microbiome and proliferate *Candida* species [10, 11]. To date, different hypotheses have been reported regarding the pathogenesis of tobacco-related diseases, but none of them conclusive. [8]..Hookah smoking is culturally popular among Asian people, however its usage becomes increasingly prevalent among other societies too [12]. Various studies reported that the rate of regular or occasional use among adults in Iran is 16.3% [13].

Consumption of opium or its derivatives as a psychoactive drug in Middle East, especially Afghanistan, Pakistan, and in our country Iran is exceptionally common [14]. A general population-based survey performed in Kerman province, Iran, indicated that the prevalence of opium abuse was 17.1% and addiction rate was 5.3% [15]. Some studies investigated the correlation between opium addiction, as a suppressive factor of immune system, and *candida* infection which showed a higher risk of fungal infection in addicts [16, 17].

Moreover, risk indicators such as cigarette, hookah and opium consumption and denture hygiene have less investigated in the previous studies. Therefor the aim of this study was to investigate the association of denture stomatitis prevalence with cigarette, hookah and opium consumption also dose–response relationship between the cigarette smoking duration and odds of denture stomatitis in population of Rafsanjan cohort center.

## Methods

### Study design and patient selection

This cross-sectional study based on data of Rafsanjan Cohort Study (RCS) which is a part of the prospective epidemiological research studies in Iran (PERSIAN) [18]. RCS conducted in Rafsanjan as one of the cities located southeast of Iran with 10,000 participants of both gender aged 35–70 years old [19]. The only inclusion criterion for the present study was wearing at least one complete denture and the exclusion criteria were as follows; immunocompromised individuals, history of drug ingestion in the past 30 days including; wide spectrum antibiotic, antimycotic, or corticosteroids and history of organ transplantation as they should receive lifelong immunosuppressive therapy. After applying the exclusion criteria, 1619 cases were included in the analysis. Study was designed based on the PERSIAN protocol and was approved by the Ethics Committee of Rafsanjan University of Medical Sciences (IR.RUMS.REC.1398.058).

### Data collection

Data were collected by completion of interviewer-based questionnaires and intra-oral examination. Intra-oral examination performed by examiners who were trained and calibrated in workshops hold by an oral medicine specialist to diagnose denture stomatitis. The oral medicine specialist was consulted in case of uncertainty. A clinical diagnosis of denture stomatitis was made when inflammatory alterations were found under a removable denture according to newton's criteria [20]. For a lesion to be classified as denture stomatitis, there must be visible inflammatory changes under the denture [21] and Patient-reported discomfort in the absence of inflammation was not recorded as denture stomatitis.

Questionnaires included questions on demography, cigarette and hookah smoking, opium consumption, alcohol consumption, medical history (diabetes and hypertension), body mass index (BMI), Wealth score index (WSI), nutritional habit and oral health questions such as denture hygiene habits. In terms of income, WSI was categorized into two groups: lower and higher than median.

To determine cigarette, hookah and opium consumption, a structured questionnaire about frequency of use and years of smoking was used. We classified cigarette smoking as current and former smoking; participants who had smoked more than 100 cigarettes in lifetime and are still smoking considered as current smoker and who quit smoking prior to admission considered as former smoker. Duration of cigarette smoking in current smokers divided into four quartiles: ≤ 11 year, 12–24 year, 25–34 year and ≥ 35 year. Opium consumption was defined as self-report of opium use at least once per week for 6 months prior to admission, and hookah smoker was defined as active smoking of tobacco using a hookah over the past 30 days [19]. Alcohol consumption was defined as drinking approximately 350 mL of beer or 150 mL of wine or 45 mL of liquor, once per week for at least 6 months[19]. Qualitative and quantitative aspects of food intake were evaluated by use of food frequency questionnaire; Vitamins A (Micrograms per day), B12 (Milligrams per day), a- and b-carotene (Micrograms per day) and calcium and magnesium (Milligrams per day) were computed by nutritional records. Questionnaires were verified in the PERSIAN [18].

### Statistical analyses

Data of the present study were analyzed by STATA 14.0 (STATA Corp, College Station, TX) software. Categorical variables across denture stomatitis were analyzed using chi-square test. T-test was used to compare the continuous variables between denture stomatitis groups. For investigating the association between cigarette, hookah and opium consumption and denture stomatitis prevalence, logistic regression models were used. Confounders were identified using relevant epidemiological literature. Thereafter, they were sequentially entered into models according to their hypothesized strengths of association with denture stomatitis. To achieve this goal, separate models at bivariate level were run to acquire variables associated with denture stomatitis. Afterwards, variables with a p-value < 0.25 were considered for multivariate analysis. Adjusted model 1 included sociodemographic characteristics (age, gender, education years and WSI) considered to be related to cigarette, hookah and opium consumption and denture stomatitis. Model 2 contained additional adjustments for oral health factor (The frequency of brushing per day), history of hypertension, alcohol use that were additionally considered to confound cigarette, hookah and opium consumption-denture stomatitis associations. Model 3 included all variables in adjusted model 2 and additionally included vitamins A, B12 and a- and b-carotene, calcium and magnesium intake per day. In all models related to cigarette smoking and denture-associated stomatitis the effect of hookah and opium use was controlled and in models related to opium consumption the unwanted effects of cigarette and hookah smoking was also controlled.

Since opium consumption is known as stigmatized and marginalized attitude, in the present study some degree of nondifferential misclassification is likely as a consequence of misreporting and recall bias. Therefore, to evaluate the direction and extent of the misreporting bias, we conducted a simple bias analysis, and compared the results with those of the conventional result [22]. The results of an internal validation analysis can be used to define the bias parameters (sensitivity and specificity of self-reported opium consumption). When resources are not available for performing an internal validation analysis, previously conducted validation studies which are applicable to the acquired data can be used. Finally, in the absence of relevant studies, the researchers must use their experience to classify parameters. Also, due to lack of any internal validation study for evaluation of the self-reporting of opium consumption, utilizing of the results of an external validation study [23] and estimation based on the current condition [19] to determine the bias parameters and applying of simple bias analysis to correct the exposure measurement error were inevitable [22].

### Results

In the present study, 1619 participants in the baseline data collection phase of the Rafsanjan cohort study, 763(47.1%) women and 856 (52.9%) men, with the mean ± SD age of 58.07 ± 7.36, who had maxillary or mandibular dentures, were included. Prevalence of denture stomatitis in all removable complete denture wearers was 21.6% (Table 1).

**Table 1:** Prevalence of Denture stomatitis in participants of the Rafsanjan Cohort Study (n = 1619)

Characteristics	All (n = 1619)	Non-denture stomatitis (n = 1270)	Denture stomatitis (n = 349)	*P*-value
**Age—year**				
Mean ± SD	58.08 ± 7.37	58.16 ± 7.38	57.79 ± 7.33	0.404
**Age—no. (%)**				
30–54	461 (28.47)	354 (27.87)	107 (30.66)	0.569
55–59	383 (23.66)	305 (24.02)	78 (22.35)	
60–64	438 (27.05)	340 (26.77)	98 (28.08)	
≥ 65	337 (20.82)	271 (21.34)	66 (18.91)	
**Gender—no. (%)**				
Female	763 (47.13)	633 (49.84)	130 (37.25)	< 0.001
Male	856 (52.87)	637 (50.16)	219 (62.75)	
**Cigarette smoking -no. (%)**				
Yes	681 (42.06)	492 (38.74)	189 (54.15)	< 0.001
No	938 (57.94)	778 (61.26)	160 (45.85)	
**Duration of Cigarette smoking—no. (%)**				
No	938 (57.94)	778 (61.26)	160 (45.85)	< 0.001
≤ 11 year	171 (10.56)	121 (9.53)	50 (14.33)	
12–24 year	174 (10.75)	126 (9.92)	48 (13.75)	
25-34 year	169 (10.44)	128 (10.08)	41 (11.75)	
≥ 35 year	167 (10.32)	117 (9.21)	50 (14.33)	
**Opium use- no. (%)**				
Yes	698 (43.11)	528 (41.57)	170 (48.71)	0.017
No	921 (56.89)	742 (58.43)	179 (51.29)	
**Hookah use- no. (%)**				
Yes	213 (13.16)	170 (13.39)	43 (12.32)	0.602
No	1,406 (86.84)	1,100 (86.61)	306 (87.68)	
**Oral health (The frequency of brushing per day)-no. (%)**				
No brushing	1,154 (71.28)	905 (71.26)	249 (71.35)	0.482
One time	192 (11.86)	156 (12.28)	36 (10.32)	
Two times	22 (1.36)	15 (1.18)	7 (2.01)	
Three times	251 (15.50)	194 (15.28)	57 (16.33)	
**WSI-no. (%)**				
Low median	807(49.87)	641 (50.47)	166 (47.70)	0.360
Over median	811(50.13)	629 (49.53)	182(52.30)	
**Education-no. (%)**				
No schooling	357(22.06)	277 (21.81)	80 (22.99)	0.178
1–5 years of school	732(45.24)	590 (46.46)	142 (40.80)	
6–12 years of school	436(26.95)	336 (26.46)	100 (28.74)	
University/college	93(5.75)	67 (5.28)	26 (4.47)	
**Alcohol use-no. (%)**				
Yes	240(14.90)	183 (14.48)	57(16.43)	0.367
No	1371(85.10)	1081 (85.52)	290(83.)	
**Hypertension-no. (%)**				
Yes	562(34.78)	466 (36.78)	96 (27.51)	< 0.001
No	1054(65.22)	801 (63.22)	253(72.49)	
**Diabetes-no. (%)**				
Yes	490(30.32)	392 (30.94)	98 (28.08)	0.304
No	1126(69.68)	875 (69.06)	253 (72.49)	
**Vitamin B12-no. (%)**				
Low median	809(50.15)	640 (50.55)	169 (48.70)	0.542
Over median	8.4(49.85)	626 (49.45)	178 (51.30)	
**Magnesium-no. (%)**				
Low median	807(50.03)	647 (51.11)	160 (46.11)	0. 099
Over median	806(49.97)	619 (48.89)	187 (53.89)	
**Calcium-no. (%)**				
Low median	807(50.03)	649 (51.26)	158 (45.53)	0.059
Over median	806(49.97)	617 (48.74)	189 (54.47)	
**Beta carotene-no. (%)**				
Low median	807 (50.03)	637 (50.32)	170 (48.99)	0.662
Over median	806 (49.97)	629 (49.68)	177 (51.01)	
**Alpha carotene-no. (%)**				
Low median	808 (50.09)	630 (49.76)	178(51.30)	0.613
Over median	805 (49.91)	636 (50.24)	169(48.70)	

Characteristics of participants from the aspects of sociodemographic, cigarette and hookah smoking, opium consumption, alcohol use, BMI, nutrient intake and oral health based on stomatitis denture status are shown in Table 1. There are significant differences regarding gender, cigarette smoking, opium consumption and history of hypertension among denture stomatitis and non-denture stomatitis participants. As expected, cigarette smoking and its duration had significant effects on prevalence of denture stomatitis.

Table 2 shows the association of denture stomatitis diseases with cigarette, hookah and opium consumption using the crude and three adjusted models.

**Table 2:** Association of cigarette, hookah and opium consumption with denture stomatitis (n = 1619)

	Crude model	Adjusted model 1	Adjusted model 2	Adjusted model 3
	OR (95%Ci)^a^	OR (95%Ci)^b^	OR (95%Ci)^c^	OR (95%Ci)^d^
**Cigarette smoking**				
No	1	1	1	1
Current	2.12 (1.63–2.76)	1.95 (1.36–2.80)	2.27 (1.52–3.38)	2.29 (1.53–3.41)
Former	1.41 (1.00–2.00)	1.29 (0.85–2.96)	1.47 (0.95–2.27)	1.46 (0.95–2.27)
**Duration of cigarette smoking**				
Not smoker	1	1	1	1
≤ 11 year	2.01 (1.39–2.91)	1.84 (1.19–2.83)	2.05 (1.30–3.23)	2.00 (1.26–3.17)
12–24 year	1.85 (1.28–2.70)	1.68 (1.08–2.63)	1.86 (1.16–2.95)	1.81 (1.13–2.90)
25–34 year	1.56 (1.05–2.30)	1.41 (0.89–2.24)	1.51 (0.92–2.47)	1.55 (0.94–2.53)
≥ 35 year	2.08 (1.43–3.02)	1.93 (1.23–3.04)	2.09 (1.29–3.36)	2.09 (1.29–3.37)
**Opium consumption**				
No	1	1	1	1
Yes	1.33 (1.05–1.69)	0.99 (0.74–1.33)	0.84 (0.61–1.17)	0.81 (0.58–1.13)
Interaction between cigarette smoking and opium use	1.45 (0.76–2.77)	1.52 (0.79–2.94)	1.51 (0.78–2.91)	1.65 (0.85–3.22)
**Hookah using**				
No	1	1	1	1
Yes	0.91 (0.64–1.30)	0.80 (0.55–1.15)	0.82 (0.56–1.19)	0.79 (0.54–1.16)

^a^The baseline model is stratified on the status of cigarette, hookah and opium consumption

^b^The adjusted model 1 is adjusted for confounding variables of age (continuous variable), gender (male/ female), education years (continuous variable) and WSI (continuous variable)

^C^The adjusted model 2 has additional adjustment for history of hypertension (yes/no), Oral health factor (e.g. brushing frequency (continuous variable), alcohol use (yes/no)

^d^The adjusted model 3 has additional adjustment for vitamins A, B12 and a- and b-carotene, calcium and magnesium (continuous variable)

In the crude regression model, the odds of denture stomatitis among current cigarette smokers and opium users were about 2.12 and 1.33 times greater than those of non-cigarette smokers and non-opium users respectively (OR; 2.12, 95% CI 1.63–2.76 and OR; 1.33, 95% CI 1.05–1.69 respectively). This association has been persisted after adjustment for confounders among current cigarette smokers (adjusted OR; 2.27, 95% CI 1.52–3.38), but not in opium users and former cigarette smokers (adjusted model 2). Adjusted model 3 includes all variables considered in adjusted model 2, additionally included vitamins A, B12 and a- and b-carotene and calcium and magnesium intake. Although, the result showed no marked changes after adjusting for the variables among current cigarette smokers (adjusted OR; 2.29, 95% CI 1.53 to 3.41) (adjusted model 3). As the results classified by quartile of cigarette smoking duration, dose–response rises were shown, with the highest odds ratios for denture stomatitis in the 4th quartile (adjusted OR; 2.09, 95% CI 1.29–3.37). Also, we investigated the interaction between opium consumption and cigarette smoking and denture stomatitis and the results showed that participants who were opium consumer and cigarette smoker at the same time had no higher odds of denture stomatitis (adjusted OR: 1.65, 95% CI 0.85–3.22). Also, we failed to find any association between hookah use and denture stomatitis in any of the models.

As previously mentioned, since opium consumption is known as stigmatized attitude in the studies, we conducted a simple bias analysis and compared the results of the analysis with conventional results, assuming that the specificity and sensitivity of the self-reported opium consumption in both denture stomatitis and non-denture stomatitis groups are 90%. The adjusted odds ratios for this bias about denture stomatitis are 1.44. These Odds ratios were reported in the crude logistics model before adjusting for this bias as 1.33. According to this analysis, percent biases, were − 7% for association between denture stomatitis and opium consumption, demonstrating that the odds ratio increased or, in other words, there was 7% error towards null prior to controlling for this bias.

## Discussion

The aim of the present cross-sectional study was to investigate the association of denture stomatitis prevalence with cigarette, hookah and opium consumption in participants of Rafsanjan cohort study, a district in southeastern Iran with a high rate of opium consumption. The present study would provide basic data for better understanding of distribution and association of denture stomatitis and cigarette, hookah and opium consumption as essential information for prevention, early diagnosis, treatment and also for better dental health services. According to our findings, the prevalence of denture stomatitis in all removable complete denture wearers was 21.6% which was in accordance with similar previous studies [4, 24]. Also In agreement with some other studies [6, 25], male participants showed a higher prevalence of denture stomatitis (62.75%). However, there are studies indicating that denture stomatitis is more frequent among female gender [24, 26].

We found that denture stomatitis were almost two times more prevalent in current cigarette smokers compared with non-cigarette smokers (also when adjusted for several potentially confounding variables). This findings were similar to those reported by several studies [5, 8, 9]. Shulman et al. found that cigarette smokers had increased OR of denture stomatitis [5]. This association could be in relation with increasing *candida* carriage, as cigarette increases epithelial thickness and changes functional activity of the keratinocytes which leads to *candida* colonization [27]. Hypothetically, cigarette smokers had a reduced salivary flow rate and, as a result, decreased saliva pH may result in an acidic environment that is also likely to increase *Candida* colonization. Furthermore, it has been proposed that smoking causes a decrease in salivary immunoglobulin A (IgA) and reduction in neutrophil function, both of which promotes oral *Candida* colonization [28]. In addition, our findings on dose–response relationships between the cigarette smoking and odds of denture stomatitis strengthened the conclusion that cigarette smoking were directly associated with an increased odds of denture stomatitis.

However, it should be noted that, the cross-sectional studies does not allow us to determine the directionality of observed associations, and further prospective studies are warranted to reconsider these associations.

Also, It has been reported that 23.81% of the Rafsanjan adults (46.19% of men and 4.27% of women) use opium [19]. Our finding indicated that the prevalence of denture stomatitis was 0.81 times lower in opium users compared to non-opium users (not statistically significant). Therefore, this result suggests that opium consumption has no effect on denture stomatitis. Interestingly, our additional analysis indicated that the interaction between opium consumption and cigarette smoking was also not associated with higher odds of denture stomatitis. Kathwate et al. observed antifungal effect of tramadol, an opioid agonist, at certain concentration [29] and in contrast to this study, Hadzic et al. [30] reported higher candida growth in opium users. There is a study suggesting that impairing immune system cells by opium can provide a *candida* infection condition and consequently increase the possibility of denture stomatitis incidence [31]. Due to these conflicting evidence, it is suggested that in the follow-up phase of the study, this association is further investigated.

According to the findings of the present work, contrary to opium users, denture stomatitis were more prevalent in hookah smokers versus non-hookah smokers which are consistent with those of the previous studies [10, 11] although they were not statistically significant.

This study had strengths and limitations. Population-based nature of the study with a large size of the sample, the high population coverage and extensive data collection for the exposure of interest (cigarette, hookah and opium consumption) and potential confounders (e.g. age, gender, education, oral heath factor, medical history and nutrient intake) were strongly enhancing the external validity of this study. The study design had a number of limitations too.

The major limitation of the study was the design where both outcome and predictor variables were assessed simultaneously. The cross-sectional design of the present study does not allow us to determine the directionality of observed associations. For example we are not able to determine if participants started smoking before or after the onset of denture stomatitis, accordingly, it is suggested that this relationship be reconsidered in the follow-up phase of this prospective study.

Also, it is likely that a number of individuals have not fully disclosed their opium consumption status. However, there might be a degree of misclassification because of self-reporting and recall biases. As a result, measurement errors may occur in this form of study, leading to incidence bias [32–34]. However, self-reporting bias whole effect varies according to sex, age, geographical location, and study population [35, 36]. Interestingly, since opium consumption in this population was related to a lower social stigma, we believe that the validity of the data on opium consumption in study population is almost high. Abnet et al. in agreement with this study showed a high rate of sensitivity of opium consumption among residents of northern cities of Iran [23]. It has been reported that this population, use opium as a traditional medicine with low social stigma. However, in the present study, findings from simple bias analysis indicated that the direction and value of this bias may be towards null and the adjusted OR for this bias about denture stomatitis is stronger than that of the conventional result and the validity of the results of this model is influenced by the accuracy of bias parameters [22]. Hence, it is suggested to perform an internal validation research on our population to examine the magnitude and direction of this bias more accurately. These findings may shed a more light in the field of causative association of denture stomatitis prevalence with opium consumption or tobacco smoking as determining factors for updating dental health services and education.

## Conclusion

According to the findings of this study while cigarette smoking has a dose–response relationship with increased odds of denture stomatitis; this association was not found with opium consumption. Also, we failed to find any association between hookah smoking and denture stomatitis. Accordingly, denture wearers should be more aware of adverse effects of cigarette smoking.

## Data Availability

The datasets used during the current study are available on the PERSIAN Adult Cohort Study Center, Rafsanjan University of Medical Sciences, Iran. The datasets generated during and analysed during the current study are not publicly available due to restriction in the ethical permission but are available from the corresponding author on reasonable request.

## Abbreviations

RCS: The Rafsanjan Cohort Study
PERSIAN: Prospective epidemiological research studies in IrAN
WSI: Wealth Score Index
BMI: Body mass index
OR: Odds ratio

## References

[1] Hilgert JB, Giordani JMdA, de Souza RF, Wendland EMDR, D'Avila OP, Hugo FN. Interventions for the management of denture stomatitis: a systematic review and meta‐analysis. J Am Geriatr Soc. 2016;64(12):2539–45. 10.1111/jgs.14399 27889906

[2] Gendreau L, Loewy ZG. Epidemiology and etiology of denture stomatitis. J Prosthodontics. 2011;20(4):251–60. 10.1111/j.1532-849X.2011.00698.x 21463383

[3] Loster JE, Wieczorek A, Loster BW. Correlation between age and gender in Candida species infections of complete denture wearers: a retrospective analysis. Clin Intervent Aging. 2016;11:1707–14. 10.2147/CIA.S116658 PMC5123722 27920509

[4] Moosazadeh M, Akbari M, Tabrizi R, Ghorbani A, Golkari A, Banakar M, et al. Denture stomatitis and Candida Albicans in Iranian Population: a systematic review and meta-analysis. J Dentistry (Shiraz, Iran). 2016;17 Suppl 3 283–92. PMC5103476 27840842

[5] Shulman JD, Rivera-Hidalgo F, Beach MM. Risk factors associated with denture stomatitis in the United States. J Oral Pathol Med. 2005;34(6):340–6. 10.1111/j.1600-0714.2005.00287.x 15946181

[6] MacEntee MI, Glick N, Stolar E. Age, gender, dentures and oral mucosal disorders. Oral Dis. 1998;4(1):32–6. 10.1111/j.1601-0825.1998.tb00252.x 9655042

[7] Vila T, Sultan AS, Montelongo-Jauregui D, Jabra-Rizk MA. Oral candidiasis: a disease of opportunity. J Fungi (Basel). 2020;6(1):15. 10.3390/jof6010015 PMC7151112 31963180

[8] Soysa NS, Ellepola AN. The impact of cigarette/tobacco smoking on oral candidosis: an overview. Oral Dis. 2005;11(5):268–73. 10.1111/j.1601-0825.2005.01115.x 16120112

[9] Alzayer YM, Gomez GF, Eckert GJ, Levon JA, Gregory RL. The Impact of Nicotine and cigarette smoke condensate on metabolic activity and biofilm formation of Candida albicans on acrylic denture material. J Prosthodontics. 2020;29(2):173–8. 10.1111/jopr.12945 30028051

[10] Mokeem SA, Abduljabbar T, Al‐Kheraif AA, Alasqah MN, Michelogiannakis D, Samaranayake LP, et al. Oral Candida carriage among cigarette‐and waterpipe‐smokers, and electronic cigarette users. Oral Dis. 2019;25(1):319–26. 10.1111/odi.12902 29800492

[11] Shakhatreh MAK, Khabour OF, Alzoubi KH, Masadeh MM, Hussein EI, Bshara GN. Alterations in oral microbial flora induced by waterpipe tobacco smoking. Int J Gen Med. 2018;11:47. 10.2147/IJGM.S150553 PMC5799848 29440924

[12] Shihadeh A. Investigation of mainstream smoke aerosol of the argileh water pipe. Food Chem Toxicol. 2003;41(1):143–52. 10.1016/s0278-6915(02)00220-x 12453738

[13] Jawad M, Charide R, Waziry R, Darzi A, Ballout RA, Akl EA. The prevalence and trends of waterpipe tobacco smoking: a systematic review. PloS one. 2018;13(2):e0192191. 10.1371/journal.pone.0192191 PMC5806869 29425207

[14] Crime. UNOoDa OWAf. 2012. Available from: https://www.unodc.org/unodc/en/data-and-analysis/WDR-2012.html.

[15] Ziaaddini H, Ziaaddini MR. The household survey of drug abuse in Kerman, Iran. JApSc. 2005;5(2):380–2.

[16] Rocchi L, D'Alessandro D, Fabiani M, Osborn J, Leonardi C, Tarsitani G, et al. P1286 Association between oral candidiasis and immunity level in a population of drug addicts in a centre for prevention and treatment of drug addictions in Rome, Italy. Int J Antimicrob Agents. 2007(29):S354–S5.

[17] Rooban T, Rao A, Joshua E, Ranganathan K. Dental and oral health status in drug abusers in Chennai, India: a cross-sectional study. J Oral Maxillofac Pathol. 2008;12(1):16–21.

[18] Poustchi H, Eghtesad S, Kamangar F, Etemadi A, Keshtkar A-A, Hekmatdoost A, et al. Prospective epidemiological research studies in Iran (the PERSIAN Cohort Study): rationale, objectives, and design. Am J Epidemiol. 2018;187(4):647–55. 10.1093/aje/kwx314 PMC6279089 29145581

[19] Hakimi H, Ahmadi J, Vakilian A, Jamalizadeh A, Kamyab Z, Mehran M, et al. The profile of Rafsanjan Cohort Study. Eur J Epidemiol. 2020:1–10. 10.1007/s10654-020-00668-7 32725579

[20] Newton A. Denture sore mouth. Brit Dent J. 1962;112:357–60.

[21] Statistics NCfH. Plan and operation of the third National Health and Nutrition Examination Survey, 1988–94: National Center for Health Statistics; 1994. 7975354

[22] Lash TL, Fox MP, Fink AK. Applying quantitative bias analysis to epidemiologic data: Springer; 2011.

[23] Abnet CC, Saadatian-Elahi M, Pourshams A, Boffetta P, Feizzadeh A, Brennan P, et al. Reliability and validity of opiate use self-report in a population at high risk for esophageal cancer in Golestan, Iran. Cancer Epidemiol Prevent Biomark. 2004;13(6):1068–70. 15184266

[24] Kaomongkolgit R, Wongviriya A, Daroonpan P, Chansamat R, Tantanapornkul W, Palasuk J. Denture stomatitis and its predisposing factors in denture wearers. J Int Dent Med Res. 2017;10(1):89–94.

[25] Kossioni AE. The prevalence of denture stomatitis and its predisposing conditions in an older Greek population. Gerodontology. 2011;28(2):85–90. 10.1111/j.1741-2358.2009.00359.x 20082642

[26] Dos Santos CM, Hilgert JB, Padilha DMP, Hugo FN. Denture stomatitis and its risk indicators in south Brazilian older adults. Gerodontology. 2010;27(2):134–40. 10.1111/j.1741-2358.2009.00295.x 19545319

[27] de Azevedo Izidoro ACS, Semprebom AM, Baboni FB, Rosa RT, Machado MAN, Samaranayake LP, et al. Low virulent oral Candida albicans strains isolated from smokers. Arch Oral Biol. 2012;57(2):148–53. 10.1016/j.archoralbio.2011.08.016 21924704

[28] Vila T, Sultan AS. Oral Candidiasis: a disease of opportunity. J Fungi. 2020;6(1):15. 10.3390/jof6010015 PMC7151112 31963180

[29] Kathwate GH, Karuppayil SM. Tramadol, an opioid receptor agonist: an inhibitor of growth, morphogenesis, and biofilm formation in the human pathogen, Candida albicans. Assay Drug Dev Technol. 2016;14(10):567–72. 10.1089/adt.2016.760 27982704

[30] Hadzic S, Dedic A, Gojkov-Vukelic M, Mehic-Basara N, Hukic M, Babic M, et al. The effect of psychoactive substances (drugs) on the presence and frequency of oral Candida species and Candida dubliniensis. Materia Socio-medica. 2013;25(4):223. 10.5455/msm.2013.25.223-225 PMC3914741 24511261

[31] Ayatollahi-Mousavi SA, Asadikaram G, Nakhaee N, Izadi A, Keikha N. The effects of opium addiction on the immune system function in patients with fungal infection. Addict Health. 2016;8(4):218. PMC5554801 28819552

[32] Gryczynski J, Schwartz RP, Mitchell SG, O'Grady KE, Ondersma SJ. Hair drug testing results and self-reported drug use among primary care patients with moderate-risk illicit drug use. Drug Alcohol Dependence. 2014;141:44–50. 10.1016/j.drugalcdep.2014.05.001 PMC4080811 24932945

[33] Yacoubian GS, Jr., VanderWall KL, Johnson RJ, Urbach BJ, Peters RJ, Jr. Comparing the validity of self-reported recent drug use between adult and juvenile arrestees. J Psychoactive Drugs. 2003;35(2):279–84. 10.1080/02791072.2003.10400010 12924751

[34] Magura S, Kang SY. Validity of self-reported drug use in high risk populations: a meta-analytical review. Substance Use Misuse. 1996;31(9):1131–53. 10.3109/10826089609063969 8853234

[35] Fendrich M, Mackesy-Amiti ME, Johnson TP. Validity of self-reported substance use in men who have sex with men: comparisons with a general population sample. Ann Epidemiol. 2008;18(10):752–9. 10.1016/j.annepidem.2008.06.001 PMC2586999 18693041

[36] Colón HM, Pérez CM, Meléndez M, Marrero E, Ortiz AP, Suárez E. The validity of drug use responses in a household survey in Puerto Rico: comparison of survey responses with urinalysis. Addict Behav. 2010;35(7):667–72. 10.1016/j.addbeh.2010.02.006 PMC2856715 20223601

